# Unveiling the gap: technical-tactical performance differences between main and qualifying draws in professional padel

**DOI:** 10.5114/biolsport.2025.150038

**Published:** 2025-04-28

**Authors:** Rafael Conde-Ripoll, Daniel Boullosa, Carlos D. Gómez-Carmona, Adrián Escudero-Tena

**Affiliations:** 1 Universidad Europea de Madrid. Department of Sports Sciences. Faculty of Medicine, Health and Sports, Madrid, Spain; 2 Faculty of Sports Sciences, University of Leon, Leon, Spain; 3 Research Group in Optimization of Training and Sports Performance (GOERD), Faculty of Sport Sciences, University of Extremadura, Caceres, Spain; 4 Research Group in Training, Physical Activity and Sports Performance (ENFYRED), Department of Music, Plastic and Body Expression, University of Zaragoza, Teruel, Spain; 5 BioVetMed & SportSci Research Group, University of Murcia, Murcia, Spain

**Keywords:** Racket sports, Performance analysis, Sex, Side of play, Efficiency, Phase, Shot type

## Abstract

This study aimed to investigate technical-tactical differences between main draw vs. qualifying draw matches in male and female professional padel players. Through systematic observation, data were collected from 39 matches (4,585 points) of the professional male qualifying draw (n = 9) and main draw (n = 13), and female qualifying draw (n = 8) and main draw (n = 9) during the 2023 World Padel Tour Finland Padel Open tournament. Using chi-square analysis and corrected standardized residuals (CSR), the results showed significant differences between draws for final shot relative to the side of the play, serving efficiency and the type of final shot. Left side male players made more forced errors (CSR = 2.8) and fewer unforced errors (CSR = 3.2) in main draw compared to qualifying draw matches. While players were in the return game, both male and female players made a higher proportion of forced errors (CSR = 3.3; CSR = 2.7) and a lower proportion of unforced errors (CSR = 3.3; CSR = 2.3) in main draw compared to qualifying draw matches. Specific shot-type analysis revealed that male and female players made varying numbers of winners, forced errors and unforced errors with certain types of shots in the main vs. qualifying draw matches. These findings provide valuable insights for players and coaches to optimize their technical-tactical strategies based on the competition draw and match context.

## INTRODUCTION

Padel is now played in 150 countries by 30 million people, making this sport one of the fastest growing sports in the world [1]. This racket sport is characterized by collaboration and opposition between players, as it is played in a doubles format [2]. The game involves dynamic ball exchanges [3] within a defined, stable playing area that measures 20 × 10 m^2^ and is surrounded by metal mesh and a wall [4], though external factors such as weather and surface conditions introduce variability in playing factors [5].

Previous research has explored the technical-tactical, physical, physiological, and psychological aspects that differentiate competitive levels in padel. Psychological factors, for instance, distinguish higher-skilled players, who exhibit greater precompetitive self-confidence and lower somatic anxiety [6]. At non-professional levels, physical attributes such as strength, power, and agility play a minimal role in performance [7], whereas among national-level players, those in the top category experience lower physical and physiological demands than their lower-division counterparts [8]. Differences also emerge in rally structure, with professional players engaging in longer rallies and executing more shots per point than regional-level players [9]. Additionally, national-level players demonstrate higher movement speed and rate of play (shots per second), while recreational players perform at lower intensities across key variables such as rally time, shots per point, distance covered, and in-play speed of movement [10]. Technical-tactical execution further distinguishes skill levels. Amateur players make more unforced errors and play fewer winners, often relying on technical-tactical actions rarely seen at higher levels (e.g., forehand and bandeja) while struggling with smashes [11]. Performance gaps extend to serve-return dynamics, as national-level players capitalize on a greater serve advantage, executing more winners and forcing opponent errors [12]. Their return strategies are also more refined, as they return low balls with greater speed and execute deeper lobs to neutralize offensive serves. Furthermore, high-level players exhibit greater tactical complexity, relying more on no-bounce shots (volley, bandeja, smash) and wall shots (back, side, doubles) to maintain control of the point [10].

Despite well-documented differences across competitive levels, research on performance variations within professional padel remains limited. Professional padel athletes primarily compete in pair-based tournaments, which form the core structure of competitive events, making the analysis of these competitions particularly relevant. These tournaments typically consist of two main sections: the qualifying draw, featuring lower-ranked teams; and the main draw, involving the top-ranked pairs along with four teams that progress from the qualifying draw. Both draws share a similar format, where prize money and ranking points increase as pairs progress through the rounds. However, to date, only one study has examined differences between qualifying and main draw players. Recently, Escudero-Tena et al. [13] found that the qualifying draw differs from the main draw in terms of rally dynamics, with more frequent net exchanges in the qualifying draw in both male and female matches.

Performance analysis in situational sports such as padel is crucial for diagnosing and enhancing technical and tactical aspects of playing [14]. Recent padel research has used notational analysis [15] to address four key areas: temporal structure [16], players’ movements [17], game scoring patterns [18], and technical-tactical actions [3]. Key findings in these areas suggest that at the professional level, on average, a point lasts approximately 9.6 shots in male matches and 12 shots in female matches [19], with rally durations ranging from 12.5 to 13.5 s regardless of sex [16]. Match length also varies as a function of sex, as over 70% of male matches conclude within 2 sets, compared to fewer than 70% in female matches [20]. Furthermore, left-side players make more winners [21, 22], execute different shots during the match, and play more shots on the partner’s side than right-side padel players [21]. In addition, winning players consistently travel greater distances and perform more accelerations per hour [17], secure more golden points [23], and make fewer unforced errors [24]. However, these insights highlight the need to explore how such performance indicators may vary across different match stages and player types, such as qualifying versus main draw players, to identify specific technical and tactical differences to adapt playing styles and strategies.

Among the four key areas of interest in performance analysis, technical-tactical actions, such as serving and overhead shots, are critical in securing points during a padel match [25, 26]. Specifically, research at professional level has demonstrated that serving provides a significant advantage in rallies under 8 shots for females and under 12 shots for males [25, 27, 28]. This advantage may be largely due to the strategic positioning of the serving pair, who move to take the net area immediately after serving, gaining a better position for securing points over the returning pair [13, 29]. Additionally, the smash has been identified as a decisive offensive shot, accounting for ~45% of winners in male and ~25% in female matches [25, 30]. However, while these recent studies have emphasized the importance of serves and smashes, there is no evidence on how these technical-tactical actions vary across different competition stages at the professional level. Understanding these variations could provide valuable insights for coaches and players in refining competitive strategies and training methods for this performance level.

Therefore, this study aims to investigate performance differences between main draw and qualifying draw matches, with focus on the effectiveness of the final shot relative to the side of play, serving efficiency, and the type of final shot in male and female professional padel players. Given the limited existing evidence on qualifying draw players, we formulated hypotheses based on established patterns in professional padel performance and expert knowledge in the sport. We hypothesized the following, irrespective of sex: (1) regardless of the playing side (right or left), main draw players would make fewer unforced errors and execute a higher number of winners and forced errors compared to qualifying draw players; (2) regardless of service efficiency (points won by servers and points won by returners), main draw players would make fewer unforced errors and execute a higher number of winners and forced errors compared to qualifying draw players; and (3) main draw players would make a higher number of winners with non-bounce shots (e.g. overhead shots and volleys) and make fewer unforced errors with bounce shots (e.g. bajadas, forehand, backhand, shots off the back wall, side wall, double wall). Through these hypotheses, we hope to shed light on the nuances of padel performance, ultimately offering practical guidance to enhance competitive play.

## MATERIALS AND METHODS

### Research design

The present observational research follows an empirical methodology with a descriptive approach, characterized by a nomothetic, punctual, and multidimensional framework [31].

### Sample

The data were collected from 39 matches (comprising 4,585 points in total) of the professional male qualifying draw (*n* = 9; including two from the first round, three from the second round, and four from the third round) and main draws (*n* = 13; including four from the first round, three from the second round, three from the quarterfinals, two from the semi-finals and one from the final), and female qualifying draw (*n* = 8; including three from the first round, three from second round, and two from the third round) and main draws (*n* = 9; including one from the first round, 3 from the second round, 2 from the quarterfinals, 2 from the semi-finals and one from the final) during the 2023 WPT Finland Padel Open tournament. All matches were played indoors in a climate-controlled arena, where the temperature was maintained at 16–18 ºC. The tournament was held between late July and early August of 2023. A total of 64 male players (34 in the qualifying draw and 30 in the main draw) and 48 female players (28 in the qualifying draw and 20 in the main draw) participated in these 39 matches. None of the main draw players analysed in the present research were either qualified from the qualifying draw or given a wild card (i.e., an invitation to participate in the tournament). All procedures were conducted according to the ethical standards in sport and exercise science research and the local Ethics Commission and following approval from the Bioethics Committee of the University of Extremadura (reference 157/2022).

### Study variables

The following variables were analysed and classified based on their core category and level of openness, where the core category represents the central thematic dimension of the variable, and the level of openness indicates the extent to which it allows variation [32].

### Independent variables

**FIG. 1:**
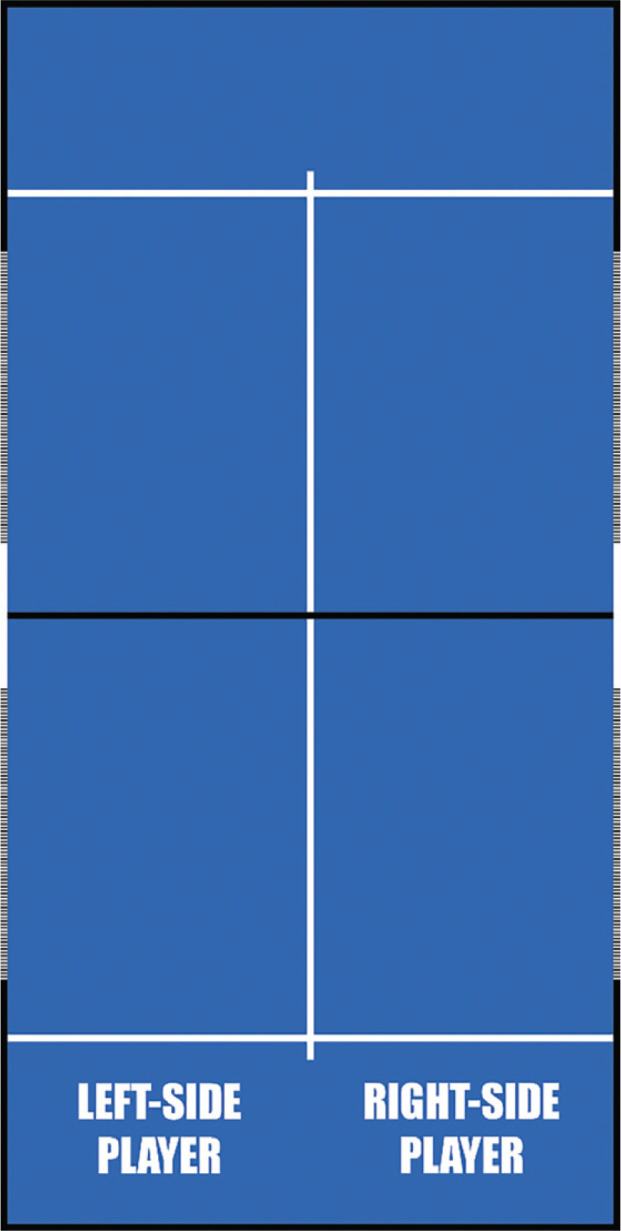
Side of play.

– Sex: it indicated whether padel players were men or women.
– Phase: a distinction was made between matches belonging to the main draw and those belonging to the qualifying draw.
– Side of play: a distinction was made between the player returning on the right side or on the left side (see Figure 1).
– Serving efficiency: a distinction was made between points won by the serving players or points won by the returning players.

### Dependent variables

– Effectiveness of the last shot (in Tables 5 and 6, this variable is treated as an independent variable): winner, forced error or unforced error. A winner can be considered as an action where a player wins the point with a direct shot, while a forced error is an action where the player loses the point due to an error in a highly difficult shot, and with a poor position for its execution due to the opponent’s prior shot. Finally, an unforced error is that action where the player loses the point due to an error made in a shot of little difficulty and with good space-time disposition for the execution [16].
– Last shot type: bandeja, smash, fake smash, recovery smash, forehand volley, backhand volley, forehand bajada, backhand bajada, forehand, backhand, back wall forehand, back wall backhand, side wall forehand, side wall backhand, double wall forehand, double wall backhand, first serve, second serve, return, contrapared and other (cadete, willy…) (see Table 1). The definition of each category was based on previous studies [25, 26].

**TABLE 1:** Last shot type and its definition

Category	Last shot type	Definition
No-bounce shots	Smash	Powerful aerial shot, typically hit between 11:30 and 1 o’clock.

	Fake smash	Aerial shot, with the same preparation as the smash, but instead of hitting it with power, before impact the player, seeking to deceive opponents, modifies the gesture so that the ball comes out slowly.

	Bandeja	Aerial shot similar to the forehand volley, with an impact range of 1:30 to 3 o’clock.

	Forehand volley	Shot made from the dominant side without a previous bounce.

	Backhand volley	Shot made from the non-dominant side without a previous bounce.

Shots off one bounce with no wall	Forehand	Shot executed after the ball bounces on the body area of the dominant side.
	Backhand	Shot executed after the ball bounces on the body area of the non-dominant side.

Shots off the wall	Recovery smash	A smash that is taken after an opponent’s power smash. That is, the opposing player makes a smash, the ball bounces on the ground and bounces on the wall(s) of the home field and then the player makes a smash.

	Forehand bajada	Offensive shot in which the ball, after bouncing on the ground and bouncing on the glass, is hit with the dominant side at chest height or above.

	Backhand bajada	Offensive shot in which the ball, after bouncing on the ground and bouncing on the glass, is hit with the non-dominant side at chest height or above.

	Back wall forehand	A shot in which the ball, after bouncing on the ground, rebounds on the back wall before being hit from the dominant side.

	Back wall backhand	A shot in which the ball, after bouncing on the ground, rebounds on the back wall before being hit from the non-dominant side.

	Side wall forehand	A shot in which the ball, after bouncing on the ground, rebounds on one of the side walls of the court before being hit from the dominant side.

	Side wall backhand	A shot in which the ball, after bouncing on the ground, rebounds on one of the side walls of the court before being hit from the non-dominant side.

	Double wall forehand	A shot in which the ball, after bouncing on the ground, rebounds on two walls, before being hit from the dominant side.

	Double wall backhand	A shot in which the ball, after bouncing on the ground, rebounds on two walls, before being hit from the non-dominant side.

Other	First serve	First service.

	Second serve	Second service.

	Return	Return of serve.

	Contrapared	A shot intentionally executed against the back or side glass of one’s own court, seeking to redirect the ball into the opponent’s court.

	Other	Any other shot that has not been defined above as a shot between the legs (willy) or any other shot not categorized.

### Procedures

The observer, a PhD student in Sports Sciences and certified padel coach with over 10 years of experience, observed the matches live and recorded the study variables through an *ad hoc* instrument. After the collection process, an intra-observer reliability analysis was performed to ensure the veracity of the data collected. The observer reanalysed a random sample of 6 matches (matches were hosted on the WPT TV website; https://www.worldpadeltourtv.com/) to ensure enough relevant data to represent 10–20% of the study sample [33]. The mean intra-observer reliability was 0.98 (see Table 2), considered almost perfect [34]. Another observer, a PhD in Sports Sciences and certified padel coach, also analysed the same random sample of 6 matches to calculate the average inter-observer reliability, which was 0.93 (see Table 2) [34].

**TABLE 2:** Inter-observer and intra-observer reliability.

Study variables	*Inter-observer*	*Intra-observer*

	K	
Sex	1.00	1.00
Phase	1.00	1.00
Effectiveness of the last shot	.84	.91
Side of play	.90	.99
Serving efficiency	.97	1.00
Last shot type	.89	.96

### Statistical analysis

Descriptive analysis of the data was performed to obtain frequency counts within each categorical variable, which we reported as both absolute numbers and percentages. Inferential tests, including Pearson’s chi-square (χ^2^), were performed to compare data categories and develop crosstab commands. This analysis verified the associations between selected variables. The strength of association between the variables was calculated using Cramer’s *V* coefficient (*Vc*) [35]. Following Crewson’s [36] recommendations for differentiating the strength of the association, based on the value, association strengths were classified as: small (< 0.100), low (0.100–0.299), moderate (0.300–0.499) or high (> 0.500). In addition, the crosstab commands made it possible to identify the associations between the categories of the variables through the corrected standardized residuals (CSR). Residuals > |1.96| revealed boxes with more or fewer cases than there should be [35]. *p* < .05 was established as the level of statistical significance, and statistical analysis was performed with IBM SPSS version 27.0 for Windows (IBM Corp., Armonk, NY).

### RESULTS

The results revealed that the phase of play is associated with the effectiveness of the last shot in left side male professional padel players (χ^2^(2) = 12.711; *p* = .002; *Vc* = 0.093) (see Table 3). Left side male players executed a higher proportion of forced errors (CSR = 2.8) and fewer unforced errors (*CSR* = 3.2) in main draw compared to qualifying draw matches.

**TABLE 3:** Comparison between main draw and qualifying draw of the effectiveness of the last shot according to the player’s side of play in men’s and women’s padel.

Effectiveness of the last shot	Men											

	Left side player						Right side player					

	Main draw			Qualifying draw			Main draw			Qualifying draw		

	n	%	CSR	n	%	CSR	N	%	CSR	N	%	CSR
Winner	391	41.8^a^	0.6	211	40.3^a^	-0.6	279	34.1^a^	0.4	145	33.0^a^	-0.4
Forced error	273	29.2^a^	**2.8**	118	22.5^b^	-2.8	267	32.6^a^	1.4	126	28.6^a^	-1.4
Unforced error	272	29.1^a^	-3.2	195	37.2^b^	**3.2**	273	33.3^a^	-1.8	169	38.4^a^	1.8

Effectiveness of the last shot	**Women**											

	**Left side player**						**Right side player**					

	**Main draw**			**Qualifying draw**			**Main draw**			**Qualifying draw**		

	**n**	%	**CSR**	**n**	%	**CSR**	**n**	%	**CSR**	**n**	%	**CSR**

Winner	162	36.2^a^	-0.3	186	37.1^a^	0.3	144	30.3^a^	0.3	130	29.4^a^	-0.3
Forced error	127	28.3^a^	1.2	125	25.0^a^	-1.2	153	32.2^a^	1.4	124	28.1^a^	-1.4
Unforced error	159	35.5^a^	-0.8	190	37.9^a^	0.8	178	37.5^a^	-1.6	188	42.5^a^	1.6

Note. n = number; % = percentage; CSR = corrected standard residuals; a, b = indicate significant differences in the Z tests for comparison of column proportions from p < .05 adjusted according to Bonferroni.

A significant relationship was found between the effectiveness of the last shot and the phase of play when points were won by the returning players in male (χ^2^(2) = 13.492; *p* = .001; *Vc* = 0.101) and female players (χ^2^(2) = 8.161; *p* = .017; *Vc* = 0.096) (see Table 4). When players are in the return game, male and female players exhibited a higher proportion of forced errors (CSR = 3.3; CSR = 2.7) and a lower proportion of unforced errors (CSR = 3.3; CSR = 2.3) in main draw compared to qualifying draw matches.

**TABLE 4:** Differences between main and qualifying draw of the effectiveness of the last shot according to the serving efficiency in men’s and women’s padel.

Effectiveness of the last shot	Men											

	Serving players						Returning players					

	Main draw			Qualifying draw			Main draw			Qualifying draw		

	n	%	CSR	n	%	CSR	N	%	CSR	N	%	CSR
Winner	462	50.1^a^	0.4	235	49.0^a^	-0.4	208	25.0^a^	0.0	121	25.0^a^	0.0
Forced error	182	19.7^a^	1.4	80	16.7^a^	-1.4	358	43.0^a^	**3.3**	164	33.9^b^	-3.3
Unforced error	278	30.2^a^	-1.6	165	34.4^a^	1.6	267	32.1^a^	-3.3	199	41.1^b^	**3.3**

Effectiveness of the last shot	**Women**											

	**Serving players**						**Returning players**					

	**Main draw**			**Qualifying draw**			**Main draw**			**Qualifying draw**		

	**n**	%	**CSR**	**n**	%	**CSR**	**n**	%	**CSR**	**n**	%	**CSR**

Winner	189	38.6^a^	0.2	188	38.1^a^	-0.2	117	27.0^a^	-0.5	128	28.5^a^	0.5
Forced error	104	21.2^a^	-0.1	106	21.5^a^	0.1	176	40.6^a^	**2.7**	143	31.8^b^	-2.7
Unforced error	197	40.2^a^	-0.1	200	40.5^a^	0.1	140	32.3^a^	-2.3	178	39.6^b^	**2.3**

Note. n = number; % = percentage; CSR = corrected standard residuals; a, b = indicate significant differences in the Z tests for comparison of column proportions from p < .05 adjusted according to Bonferroni.

The analysis also identified a significant relationship between the type of the last shot and the phase of play when the last shot was an unforced error in male players (χ^2^(16) = 28.368; *p* = .029; *Vc* = 0.177) (see Table 5). Male players made more winners with the recovery smash (CSR = 2.1) in the main draw, while they performed more winners with the side wall backhand (CSR = 2.4) in the qualifying draw. Additionally, male players executed more forced errors with the back wall backhand (CSR = 2.2) in qualifying draw matches. Furthermore, male players made more unforced errors with the bandeja (CSR = 2.0) and forehand volley (CSR = 2.2) in the main draw, while they made more unforced errors with the forehand (CSR = 2.9) in the qualifying draw.

**TABLE 5:** Differences between main draw and qualifying draw according to the effectiveness and type of the last shot in men’s padel

Shot family	Shot type	Winners				Forced errors				Unforced errors			

		M		Q		M		Q		M		Q	

		%	CSR	%	CSR	%	CSR	%	CSR	%	CSR	%	CSR
Without bounce	Smash	44.3^a^	-0.1					44.7^a^	0.1	5.3^a^	-0.3	5.8^a^	0.3
	Bandeja	13.4^a^	1.0	11.2^a^	-1.0	0.2^a^	0.7	0.0^a^	-0.7	22.4^a^	**2.0**	17.0^a^	-2.0
	Forehand volley	14.2^a^	0.4	13.2^a^	-0.4	10.6^a^	-0.6	11.9^a^	0.6	17.4^a^	**2.2**	12.1^a^	-2.2
	Backhand volley	12.4^a^	-0.9	14.3^a^	0.9	24.1^a^	0.7	21.7^a^	-0.7	12.1^a^	-1.1	14.6^a^	1.1

With bounce and without wall	Forehand	2.2^a^	-1.3	3.7^a^	1.3	7.4^a^	-0.6	8.6^a^	0.6	9.5^a^	-2.9	15.9^a^	**2.9**
	Backhand	1.6^a^	-0.1	1.7^a^	0.1	8.9^a^	-0.1	9.0^a^	0.1	12.8^a^	-0.9	14.8^a^	0.9

With bounce and with wall	Forehand bajada	1.6^a^	-1.3					2.8^a^	1.3	6.4^a^	1.2	4.4^a^	-1.2
	Backhand bajada	0.4^a^	1.3	0.0^a^	-1.3	0.0^a^	-1.5	0.4^a^	1.5	0.7^a^	0.9	0.3^a^	-0.9
	Back wall forehand	0.6^a^	-0.5	0.8^a^	0.5	11.3^a^	0.8	9.4^a^	-0.8	4.2^a^	-1.6	6.6^a^	1.6
	Back wall backhand	0.9^a^	-0.4	1.1^a^	0.4	6.9^a^	-2.2	11.5^b^	**2.2**	3.7^a^	1.3	2.2^a^	-1.3
	Out of the court	1.0^a^	0.3	0.8^a^	-0.3	2.2^a^	1.4	0.8^a^	-1.4	0.2^a^	0.8	0.0^a^	-0.8
	Side wall forehand					2.8^a^	-0.1	2.9^a^	0.1	0.9^a^	-0.6	1.4^a^	0.6
	Side wall backhand	0.0^a^	-2.4	0.8^b^	**2.4**	3.9^a^	0.4	3.3^a^	-0.4	2.4^a^	-0.1	2.5^a^	0.1
	Double wall forehand	1.3^a^	-0.1	1.4^a^	0.1	5.7^a^	-0.4	6.6^a^	0.4	1.3^a^	0.7	0.8^a^	-0.7
	Double wall backhand	0.9^a^	0.6	0.6^a^	-0.6	6.9^a^	0.6	5.7^a^	-0.6	0.7^a^	-0.2	0.8^a^	0.2

	Recovery smash	4.6^a^	**2.1**	2.0^b^	-2.1	0.7^a^	-0.1	0.8^a^	0.1

Other	Contrapared					6.3^a^	-0.1	6.6^a^	0.1	0.0^a^	-1.2	0.3^a^	1.2
	Serve	0.3	-1.2	0.8	1.2	0.0^a^	-1.7	0.5^a^	1.7
	Other					2.2^a^	1.4	0.8^a^	-1.4				

Note. M = main draw; Q = qualifying draw; n = number; % = percentage; CSR = corrected standard residuals; CSR > 1.96: bold; a, b = indicate significant differences in the Z tests for comparison of column proportions from p < .05 adjusted according to Bonferroni.

Finally, the results showed a significant relationship between the type of the last shot and the phase of play when the last shot was a winner (χ^2^(13) = 0.058; *p* = .058; *Vc* = 0.187) and an unforced error in female padel (χ^2^(16) = 29.837; *p* = .019; *Vc* = 0.204) (see Table 6). Female players made more winners with the recovery smash (CSR = 2.0) in the main draw, while they performed more winners with the back wall backhand (CSR = 2.3) in the qualifying draw. Additionally, female players executed more forced errors with the side wall backhand (CSR = 2.0) in the qualifying draw matches. Furthermore, female players made more unforced errors with the forehand bajada (CSR = 3.6) and backhand bajada (CSR = 2.1) in the main draw, while they made more unforced errors with the smash (CSR = 2.2) in the qualifying draw.

**TABLE 6:** Differences between main draw and qualifying draw according to the effectiveness and type of the last shot in female padel

Shot family	Shot type	Winners				Forced errors				Unforced errors			

		M		Q		M		Q		M		Q	

		%	CSR	%	CSR	%	CSR	%	CSR	%	CSR	%	CSR
Without bounce	Smash	26.5^a^	0.8	23.7^a^	-0.8					2.7^a^	-2.2	6.1^b^	**2.2**
	Bandeja	21.6^a^	0.5	19.9^a^	-0.5	0.0^a^	-1.1	0.4^a^	1.1	27.0^a^	0.2	26.5^a^	-0.2
	Forehand volley	21.6^a^	0.3	20.6^a^	-0.3	10.0^a^	0.1	9.6^a^	-0.1	13.4^a^	-1.1	16.4^a^	1.1
	Backhand volley	14.4^a^	0.7	12.3^a^	-0.7	21.4^a^	0.7	18.9^a^	-0.7	11.3^a^	-0.6	12.7^a^	0.6

With bounce and without wall	Forehand	3.6^a^	-1.4	6.0^a^	1.4	6.4^a^	-1.2	9.2^a^	1.2	9.2^a^	-1.0	11.4^a^	1.0
	Backhand	2.0^a^	-2.1	5.1^a^	2.1	9.6^a^	-1.0	12.4^a^	1.0	11.3^a^	0.6	9.8^a^	-0.6

With bounce and with wall	Forehand bajada	4.6^a^	-0.3	5.1^a^	0.3					8.9^a^	**3.6**	2.6^b^	-3.6
	Backhand bajada	0.0^a^	-1.4	0.6^a^	1.4					1.8^a^	**2.1**	0.3^b^	-2.1
	Back wall forehand	2.6^a^	0.9	1.6^a^	-0.9	8.6^a^	-0.4	9.6^a^	0.4	5.6^a^	0.9	4.2^a^	-0.9
	Back wall backhand	0.7^a^	-2.3	3.2^b^	**2.3**	8.9^a^	-0.1	9.2^a^	0.1	2.1^a^	-0.9	3.2^a^	0.9
	Out of the court	1.4^a^	1.9	0.0^a^	-1.9
	Side wall forehand					5.7^a^	1.4	3.2^a^	-1.4	1.5^a^	-0.4	1.9^a^	0.4
	Side wall backhand					2.5^a^	-2.0	6.0^b^	**2.0**	3.0^a^	0.7	2.1^a^	-0.7
	Double wall forehand	0.0^a^	-1.4	0.6^a^	1.4	11.4^a^	1.1	8.4^a^	-1.1	0.6^a^	-0.3	0.8^a^	0.3
	Double wall backhand	0.7^a^	-0.4	0.9^a^	0.4	7.5^a^	-0.4	8.4^a^	0.4	1.2^a^	-0.7	1.9^a^	0.7
	Recovery smash	1.3^a^	**2.0**	0.0^a^	-2.0	0.0^a^	-1.1	0.4^a^	1.1
Other	Contrapared					5.4^a^	0.7	4.0^a^	-0.7	0.3^a^	1.1	0.0^a^	-1.1
	Serve	0.7^a^	0.6	0.3^a^	-0.6					0.0^a^	-0.9	0.3^a^	0.9
	Other					1.1^a^	1.6	0.0^a^	-1.6				

Note. M = main draw; Q = qualifying draw; n = number; % = percentage; CSR = corrected standard residuals; CSR > 1.96: bold; a, b = indicate significant differences in the Z tests for comparison of column proportions from p < .05 adjusted according to Bonferroni.

## DISCUSSION

This study aimed to examine performance differences between main draw and qualifying draw players, focusing on the effectiveness of the final shot relative to the side of play, serving efficiency, and the type of final shot in both male and female professional padel players. This investigation addresses a significant gap in padel research, as understanding performance variations across competition levels is essential for players’ development and coaching strategies. The results revealed distinctive patterns between the two competition stages across specific contextual variables which are discussed in light of the proposed hypotheses.

The first hypothesis suggested that, regardless of the side of play (right or left), players in the main draw would make fewer unforced errors and play more winners and forced errors compared to players in the qualifying draw. The findings partially support this hypothesis. Male players on the left side in main-draw matches made fewer unforced errors and more forced errors compared to their counterparts in the qualifying draw. However, no significant differences were observed among female players, regardless of the side of play, or among male players on the right side between the main and qualifying draws. These findings are novel, as no prior study has conducted such a detailed analysis of these variables in padel, presenting both a unique opportunity and a challenge for contextualization.

Previous research on men’s padel has shown that left-side players play more winners [21, 22], perform distinct technical-tactical actions throughout the match, and hit more shots on their partner’s side compared to right-side players [21]. Other studies in padel have identified differences across competition levels [9] and between sexes [26]. Collectively, these findings highlight sex as a key contextual variable influencing the effectiveness of the final shot across competition stages. Therefore, while no significant differences were observed for female players or male players on the right side between main and qualifying draws, male players on the left side should focus on reducing unforced errors in qualifying matches. In contrast, in main-draw matches, they should develop strategies to better manage pressure situations to minimize forced errors. These insights provide actionable recommendations for tailoring coaching interventions and player preparation based on the specific demands of competition stages and playing contexts.

The second hypothesis suggested that, regardless of service effectiveness (points won by servers and points won by returners), players in the main draw would make fewer unforced errors and a greater number of winners and forced errors compared to players in the qualifying draw. However, the results revealed differences only in points won by returners. Specifically, both male and female players made more forced errors and fewer unforced errors in main-draw matches compared to qualifying-draw matches.

These findings are particularly novel, as no previous studies in padel have combined the variables of service effectiveness, final shot effectiveness, sex, and competition stage in a single analysis. Nonetheless, some prior research has examined service effectiveness alongside various performance parameters in padel, revealing differences that align with the results of this study [18, 27, 37]. These results suggest that service effectiveness may influence the performance of the final shot, depending on whether players are competing in the main draw or the qualifying draw. While no significant differences were found in points won by servers between competition stages, the results for points won by returners carry important practical implications. For players of both sexes competing in the qualifying draw, it is crucial to focus on enhancing tactical decision-making and improving technical execution to reduce unforced errors. In contrast, players preparing for main-draw matches should prioritize training under conditions of pressure or disadvantage to minimize forced errors during rallies. These findings emphasize the need for tailored training programmes that address the specific demands of competition stages, enabling players to develop both tactical and technical skills suited to the challenges of main-draw and qualifying-draw matches. Finally, numerous studies have examined the typology of the final shot in professional male and female padel, highlighting differences in its effectiveness [25, 26]. However, no prior research has differentiated performance by competition stage at the professional level.

Our final hypothesis proposed that players in the main draw would play a higher number of winners with shots taken without a bounce (e.g., overheads and volleys) and make fewer unforced errors with groundstrokes (shots with a bounce, such as forehands, backhands, and those following a rebound off the back wall, side wall, or double wall). Contrary to this hypothesis, the results indicate that in the main draw, both male and female players made more winners with recovery smashes and made more unforced errors with specific shots: the bandeja (males), forehand volleys (males), and descending forehand and backhand groundstrokes (females). Conversely, in the qualifying draw, males made more winners with backhand groundstrokes off the side wall, while females made more winners with backhand groundstrokes off the back wall. Regarding errors, males made more forced errors with backhand groundstrokes off the back wall, and females made more forced errors with backhand groundstrokes off the side wall. Additionally, males made more unforced errors with forehands, while females made more unforced errors with smashes. Overall, these findings suggest that success in the main draw is closely associated with the effectiveness of offensive shots, such as overheads, volleys, and bajadas. In contrast, in the qualifying draw, the effectiveness of defensive shots (e.g., backhand groundstrokes off the back and side walls, forehands) appears to play a more decisive role, along with the smash for female players.

These insights underscore the importance of tailoring technical and tactical training for professional players depending on their level. For main-draw players, emphasis should be placed on refining offensive shot execution, while qualifying-draw players may benefit from strategies aimed at improving defensive shot consistency and accuracy. Similar patterns have been observed in other racket sports. In pickleball, the left-side player of the winning team performs more shots per game, indicating a dominant role in point construction [38]. In men’s doubles tennis, elite teams make few unforced errors during the match, with forced errors being the primary means of point conclusion [39]. Furthermore, experienced teams require fewer shots and less time to win points than newer teams [40]. These similarities among racket sports suggest that competitive level influences match dynamics across various doubles formats, reinforcing the need for sport-specific yet transferable training strategies.

## Limitations and future studies

Although this study provides valuable insights into technical-tactical differences between competition levels in professional padel, offering an evidence-based framework for coaches and players, some limitations should be acknowledged. Data were collected from a single tournament, which might not fully represent performance patterns across the entire professional circuit, and environmental conditions and court characteristics specific to this tournament could have influenced playing patterns. Based on these limitations, future research should expand these findings across multiple tournaments in different contexts. Additionally, investigating the relationship between technical-tactical patterns and physical demands across competition levels could provide valuable insights for training prescription. Longitudinal studies tracking players’ progression from qualifying to main draws would enhance our understanding of performance evolution and facilitate the development of targeted interventions for competitive advancement. In addition, the current study did not include a direct analysis of fatigue-related variables such as match duration, energy expenditure, or physical performance metrics. Therefore, future studies should investigate how fatigue affects performance across different stages of a tournament, including its potential role in the observed technical-tactical differences.

## CONCLUSIONS

This study identified key performance differences between the main draw and qualifying draw in professional padel, suggesting that when both competing teams are at a higher level, the demands of the game change. Specifically, male players on the left side made more forced errors and fewer unforced errors in main-draw matches compared to qualifying-draw matches. Additionally, when players are in the return game, both male and female players made more forced errors and fewer unforced errors in main-draw matches than in qualifying-draw matches. These patterns suggest a higher level of tactical decisionmaking and technical execution in main-draw matches, where players aim to force errors from their opponents rather than relying on unforced errors.

The analysis of shot types revealed distinct patterns based on the competition stage. In the main draw, recovery smashes were associated with a higher number of winners for both men and women, while unforced errors were more frequent with the bandeja (men), forehand volleys (men), and forehand and backhand bajadas (women). In the qualifying draw, winners were more commonly achieved with backhand groundstrokes off the side wall (men) and off the back wall (women). Forced errors were more frequent with backhand groundstrokes off the back wall (men) and the side wall (women), while unforced errors were more common with forehands (men) and smashes (women).

From a practical perspective, these findings provide valuable insights for players, particularly those aspiring to compete in the main draw. Coaches can use these insights to design training protocols that refine specific technical and tactical skills according to phase competition demands. For players preparing for the main draw, emphasis should be placed on mastering recovery smashes, optimizing shot selection under pressure, and improving consistency in fast-paced exchanges. In contrast, preparation for the qualifying draw should focus on reducing unforced errors, improving consistency in wall-based shots, and developing more aggressive playing patterns. Understanding these performance differences can help players and coaches tailor training sessions to better meet the demands of high-level competitions.

## Data Availability

Data will be available upon request.
